# Navigation Techniques in Endoscopic Spine Surgery

**DOI:** 10.1155/2022/8419739

**Published:** 2022-08-29

**Authors:** Matthew J. Hagan, Thibault Remacle, Owen P. Leary, Joshua Feler, Elias Shaaya, Rohaid Ali, Bryan Zheng, Ankush Bajaj, Erik Traupe, Michael Kraus, Yue Zhou, Jared S. Fridley, Kai-Uwe Lewandrowski, Albert E. Telfeian

**Affiliations:** ^1^Warren Alpert School of Medicine of Brown University, 222 Richmond Street, Providence, RI 02903, USA; ^2^Department of Neurosurgery, CHR Citadelle, Bd du 12eme de Ligne, 1, 4000 Liege, Belgium; ^3^Department of Neurosurgery, Warren Alpert School of Medicine of Brown University, 593 Eddy Street, APC 6, Providence, RI 02903, USA; ^4^Helios Weißeritztal Clinics, Bürgerstraße 7, 01705 Freital, Germany; ^5^ORTHix Zentrum für Orthopädie, Stadtberger Str. 21, 86157 Augsburg, Germany; ^6^Department of Orthopaedics, Xinqiao Hospital, Third Military Medical University, Chongqing 400037, China; ^7^Center for Advanced Spine Care of Southern Arizona, The Surgical Institute of Tucson, 4787 E Camp Lowell Dr, Tucson, AZ 85712, USA

## Abstract

Endoscopic spine surgery (ESS) advances the principles of minimally invasive surgery, including minor collateral tissue damage, reduced blood loss, and faster recovery times. ESS allows for direct access to the spine through small incisions and direct visualization of spinal pathology via an endoscope. While this technique has many applications, there is a steep learning curve when adopting ESS into a surgeon's practice. Two types of navigation, optical and electromagnetic, may allow for widespread utilization of ESS by engendering improved orientation to surgical anatomy and reduced complication rates. The present review discusses these two available navigation technologies and their application in endoscopic procedures by providing case examples. Furthermore, we report on the future directions of navigation within the discipline of ESS.

## 1. Introduction

Across surgical specialties, there has been a paradigm shift from open procedures towards minimally invasive techniques. This procedural transition is especially true for the field of neurosurgery—including spine surgery. Many procedures that once employed open surgical approaches now utilize minimally invasive techniques that leverage technologies such as endoscopy. Endoscopic spine surgery (ESS), encapsulated by the discipline of minimally invasive spine surgery (MISS), utilizes small incisions through which endoscopes are inserted to visualize the surgical field. Endoscopic approaches generally leverage natural anatomical corridors to gain access to spinal pathology in an ultraminimally invasive fashion. They have been successfully employed to accomplish various spinal procedures across the cervical, thoracic, and lumbar spine [1]. These procedures include but are not limited to discectomies, decompressions, and fusions [1, 2]. The two most common ESS approaches to percutaneous endoscopic lumbar surgery (PELS) are transforaminal PELS (tPELS) and interlaminar PELS (iPELS). In the present narrative review article, the authors discuss the history, feasibility, advantages, disadvantages, and future directions of navigation's use in endoscopic and minimally invasive spine surgeries.

An endoscopic approach affords multiple benefits over other surgical approaches to the spine. The use of incisions less than one inch in length attenuates the degree of tissue trauma and blood loss during the procedure [3]. In addition, these minimal incisions prevent the need for extensive lamina or facet resection and dural sac retraction leading to reduced epidural fibrosis and iatrogenic instability [3, 4]. Furthermore, the combination of local anesthetic use, conscious sedation, and reduced need for postoperative medications engender shorter hospitalization and faster recovery [3, 5]. Further, shorter hospitalization times and the possibility of same-day surgeries may allow for higher patient throughput while minimizing costly inpatient stays [6].

Unfortunately, ESS has a steep learning curve when coupled with conventional fluoroscopic guidance. ESS presents two distinct challenges to surgeons attempting to adopt the technique. First, it may be challenging to target the spinal pathology and confirm that the diseased areas have been addressed. Second, surgical anatomy visualized through the endoscope may be unfamiliar. Navigation, via optical or electromagnetic mediums, potentially affords the spine surgeon superior localization of the pathology and intraoperative confirmation of pathology resolution. The use of navigation may allow for easier adoption by trainees who will likely use ESS in their future practices given its growing popularity and range of indications [1, 7]. In addition, for tPELS, optimal positioning of the working channel is crucial to avoid operative complications such as injury to the exiting nerve root or blood vessels and to treat foraminal stenosis or herniated discs effectively [8]. Radiation dose can increase dramatically for the patient and the medical staff when multiple fluoroscopies are needed to obtain optimal positioning of the working channel [8]. For these reasons, more recently developed intraoperative navigation techniques have been readily applied to ESS.

## 2. Available Navigation Technologies and Their Application to Endoscopic Spine Surgery

### 2.1. History of Navigation in Spine Surgery

Sensitive neurovascular structures and considerable anatomic complexity within the spine have stimulated the development of technologies to improve the safety and accuracy of spinal surgery. Historically, a free-hand approach to pedicle screw placement was used. However, this technique can lead to misplaced screws and revision surgeries, promoting the development of advanced intraoperative imaging to guide screw placement [9–13]. In 1981, one of the first case series was published employing intraoperative fluoroscopic guidance to decompress the cervical spine [14]. Advent of C-arm imaging allowed for more accurate pedicle screw placement by allowing the surgeon to visualize patient bony anatomy on two-dimensional (2D) fluoroscopic imaging [15]. O-arm imaging further built upon this technique, permitting three-dimensional (3D) images to be cast instantaneously on a monitor obviating the requirement to account for patient positioning [15]. More recently, computer-assisted navigation (CAN) allows for real-time reconstruction of an interactive 3D map that synchronizes patient anatomy and the operator's guided instruments. In 1995, the first reports of CAN aiding pedicle screw placement were published [16, 17]. Since this time, CAN technology has gained widespread adoption, and the number of systems has increased [18]. Today, CAN afford even more precise pedicle screw placement with the advent of modern adjuncts such as intraoperative CT scanners, stereotactic 3D cameras, reference arrays, and navigation software [19–23]. The advancement of on-skin trackers may prevent the need for traditional reference arrays, which must be secured to the patient's bony anatomy and obstruct the surgical field [22]. Concurrently with technological navigation innovation, advances in EM navigation [24, 25], robotic guidance [18], and assistance through augmented reality [26] have also been developed and promise even further near-term advances in the utility of these systems, as further described below.

### 2.2. Optical Systems

There are two main types of intraoperative navigation systems: optical and electromagnetic. Optical systems use an infrared tracking camera, such as intraoperative CT-guided navigation (ICT-Nav). A computed tomography (CT) scan of the patient is synchronized with an optical navigation system utilizing a mounted reference array that is physically attached to an element of the patient's bony anatomy, often a spinous process or posterior superior iliac spine. The intraoperative CT scan is acquired after the reference array has been secured to the patient, which allows for real-time visualization of the navigated surgical instruments in reference to patient anatomy.

### 2.3. Electromagnetic Systems

Electromagnetic navigation works with a control unit, an electromagnetic field generator, and specialized sensors that can be detected when moving within the electromagnetic field [27]. An electromagnetic field generator, a monitor, and sensors are needed to navigate this system's instruments. Sensors are attached to the patient (“patient trackers”) and the instruments. The generator creates an electromagnetic field close to the desired working area and can identify the relative position of the sensors. Those sensors can be placed at the tip of the navigated instruments.

### 2.4. Utility of Navigation Technologies in Endoscopic Spine Surgery

Traditionally, MISS relies on fluoroscopic imaging guidance to direct retractors, localize instruments, and monitor progress in surgical procedures like discectomy and decompression. Similar to its use in open procedures, fluoroscopy allows for safe and efficient access to the neuroforamen. 2D fluoroscopy in MISS has inherent disadvantages. These shortcomings include insufficient information on the completeness of bony decompression and increased radiation exposure to both the surgeon, operating room staff, and patient [28]. 2D fluoroscopy does not provide real-time imaging guidance, necessitating the operating room staff to pause an operation and possibly step away from the surgical field to obtain images. This interrupted image capture may increase procedure duration and may result in less accurate navigation as compared to other techniques [24, 25, 29, 30].

Optical systems are well-established as a safe and effective alternative to fluoroscopic guidance while offering improved accuracy of pedicle screw placement in MIS [31, 32]. The use of ICT-Nav in spine surgery has demonstrated high accuracy of pedicle screw placement, avoidance of intraoperative errors, and reduced need for revision surgeries [20, 21, 33–35]. In a recent meta-analysis, Li et al. described a final pedicle screw accuracy rate of 92.7%. Other researchers have noted improved pedicle screw placement accuracy as compared to fluoroscopy [36]. Furthermore, ICT-Nav may reduce the need for intraoperative and postoperative pedicle screw revisions. Habib et al. reported that 3.0% of pedicle screws required intraoperative revision [20], while Hecht et al. in their cohort of 260 patients, only 1 (0.5%) patient required postoperative revision surgery [34]. Additionally, ICT-Nav may also decrease radiation exposure staff risk to develop cataracts or even solid tumors to the operating staff [37–39]. Understanding the relationship between navigation and radiation is imperative in MIS. Studies have shown that MIS surgeons may be at heightened risk compared to more invasive techniques that rely less on image guidance [40].

The potential benefits of ESS paired with navigation are multiple. CAN has been primarily used in conventional open surgical approaches. Still, it has also been increasingly utilized in MIS. In ESS, the operative field is smaller, and anatomy appreciation through the endoscope can be complex. ICT-Nav allows for improved radiographic visualization of bony and soft tissue anatomy adjacent to the trajectory of approach thanks to the application of CT while maintaining tiny incisions. These capabilities have allowed spine surgeons to apply computer-assisted MIS for a myriad of procedures, including but not limited to pedicle screw fixation, transforaminal lumbar interbody fusion (TLIF), lateral/oblique lumbar interbody fusion (LLIF/OLIF), and vertebroplasty/kyphoplasty [1, 41]. Improved pedicle screw accuracy using ICT-Nav as compared to fluoroscopy has been replicated in MIS as well [42]. Furthermore, percutaneous navigation may lead to reduced facet violation rates compared to navigated open procedures [43].

CAN may improve the learning curve of MISS and endoscopic spine surgery procedures. As CAN becomes widely adopted for open procedures, both trainees and experienced surgeons will become accustomed to computer-navigated surgery workflow and techniques. These abilities will likely translate to its use in ESS. As discussed, ESS has a steep learning curve due to the small fields of vision, disorienting surgical anatomy under the endoscope, and limited resolution of the endoscopic images. Like navigation adoption in open procedures, the early experience can be improved over time as CAN may present more familiar anatomic representations via a virtual medium. The learning curve of CAN for traditional open spine procedures flattens at six months and plateaus at 12 months [44]. These results have been replicated in MIS approaches to transpedicular screw placement procedures [45]. Wood and McMillen found that as surgeons gained experience with navigation use in MISS for pedicle screw placement, intraoperative pedicle screw malposition rates decreased with time [45]. Specifically, the authors analyzed consecutive minimally invasive lumbar fusions over four years and found that rates of screw malposition decreased from 5.1% for the first 50 patients to 2.1% for the last 50 patients [45]. Fan et al. (2016) expanded these findings, concluding that navigation improves the learning curve in surgeons learning transforaminal percutaneous endoscopic lumbar discectomy [4]. Particularly, the authors found that navigation assistance may improve trainee fluoroscopy time, preoperative location time, puncture-channel time, and overall operation time when performing the procedure [4]. Research investigating electromagnetic navigation in other disciplines has shown that operation time stabilizes after performing additional procedures [46]. For example, Schnurr et al. found that orthopedic residents implant their first total knee arthroplasty with the same degree of accuracy as experienced surgeons when using computer navigation [47]. However, the authors reported that the more inexperienced group had prolonged operation times [47]. Nousiainen et al. reported that surgical trainees learning to place screws to correct femoral neck fractures took fewer attempts and had less fluoroscopy time compared to those trained using conventional fluoroscopic techniques [48].

Presently, only case reports [49] and small case series [50–53] have been published on the feasibility of CAN within ESS. Shin et al. (2020) reported 23 patients who underwent ESS using a hybrid approach employing multiaxis robotic C-arm with an image-guided navigation system. Their patient cohort was comprised of 17 patients that underwent lumbar surgery and six patients who had a cervical spine procedure. The authors reported high-quality clinical outcomes without surgical complications [50]. Nonetheless, there is a lack of literature comparing the relative effectiveness of navigation within endoscopic spine surgery against nonnavigated procedures [1]. As a result, future research comparing navigated versus nonnavigated approaches in ESS across a range of indications is needed; primary outcomes must include clinical benefit and radiation exposure to the operating room staff and patients, as well as cost.

As alluded to above, shortcomings of optical systems include line-of-sight issues between the surgical field and the tracking camera, given that the reflective marker spheres need to be directly visualized to maintain navigation. In addition, the marker spheres are typically attached to the handle of the instruments, and therefore flexible needle tips cannot be reliably navigated [54]. Moreover, if the reference array moves during the procedure, this may necessitate rescanning the patient to recalibrate the system, contributing to additional radiation exposure to the patient and the medical staff. Specific to navigation within MIS surgery, bony landmarks may not be as visible. As a result, incorrect positioning of the navigated instruments may not be as apparent to the operator. Similarly, the adoption of navigation for open techniques has a steep learning curve regarding optimal workflow.

EM navigation may afford unique advantages as compared to conventional navigation techniques as well as optical CAN. EM navigation has been shown to have similar accuracy as compared to optical navigation procedures while obviating the line-of-sight limitation inherent to optical systems [25]. The requisite clear line-of-sight in optical systems restricts the operator's degree of freedom [24]. More freedom with movement and trainees not having to worry about line of site issues may allow them to focus more on the critical parts of ESS procedures improving the learning curve.

Wu et al. (2020) investigated the utility of EM navigation compared to conventional fluoroscopy for transforaminal endoscopic lumbar decompression in a randomized control trial [25]. The researchers found that procedure time and cannula placement time were significantly faster in the EM navigation group. In addition, radiation dose was less in the EM group, and functional outcomes were similar between the two groups [25]. Yao et al. (2021) reported similar findings in their retrospective case series of 29 patients and 162 screws reporting faster operative time, less radiation time, and more precise pedicle screw placement time in the EM navigation group as compared to conventional fluoroscopy [24]. Improved accuracy and efficiency of EM-based navigation MISS procedures may also lead to improved patient outcomes [24, 55]. It has been proposed that a potential reason for improved EM outcomes as compared to fluoroscopy is that EM allows for real-time intraoperative multiplanar visualization. In addition, EM navigation may improve the learning curve of MISS as the system allows for the automatic alignment of the patient anatomy based off preoperative CT affording quick and easy set-up [24]. Operative time optimization using EM may also be a function of improving the efficiency of guide wire and working tube positioning through the safe triangle of the spinal canal—a particularly difficult part for beginners [55, 56].

EM navigation also allows for trainees to learn MISS on a broader range of instruments and pathologies thus further improving the learning curve. EM-based navigation allows for the use of flexible instruments, such as puncture needles, endoscopes, and catheters, as well as expandable application use for percutaneous procedures under EM guidance [24, 25]. Broadening the instruments a trainee can potentially use allows them to choose those they feel most comfortable with as well as developing a more well-rounded surgeon. Lastly, the use of EM navigation allows for guidance with a small device, namely, the Access Tracker. When coupled with the registration device, the IseeTracker, the system enables for precise visualization of complex spine anatomy including intervertebral collapse, severe hyperosteogeny, and revision surgeries [55]. Application MIS techniques to these more complex cases allow trainees to learn on a myriad of pathologies potentially further improving the learning curve [55].

In general, one of the significant drawbacks of the tPELS technique guided by fluoroscopy alone without navigation is the need to obtain repeat intraoperative X-rays to perfectly position the working channel in a trial-and-error manner, especially for beginners [4]. This increases operative time, and the staff risks developing cataracts or even solid tumors due to increased exposure to radiation [54]. Repeated punctures while finding the optimal channel this way may also lead to nerve root, vessels, or bowel injuries [57]. While optical systems also mitigate this, they are also associated with radiation exposure since intraoperative CT scans must be obtained. A growing body of evidence suggests electromagnetic navigation, which requires only a pair of fluoroscopy images at the start of the registration process, can dramatically reduce irradiation for the staff and for the patient, as well as the number of puncture trials needed to position the working channel perfectly [25, 55]. Navigation of the entry needle has also been associated with a flattening of the beginner's learning curve for the tPELS technique [8].

Disadvantages to electromagnetic navigation in ESS are limited. The major drawback of the electromagnetic navigation technique is the perturbation of the sensors' localization when ferromagnetic objects are present within the electromagnetic field. The registration process can be more time-consuming than optical navigation and prolong overall operative time. In addition, to our knowledge, the cost-effectiveness of electromagnetic navigation in PELS has yet to be demonstrated.

CAN and EM may allow for better localization of instruments related to the operative site throughout the procedure, avoiding potential injury [24, 55, 56, 58]. This ability may or may not further the skills of an experienced surgeon performing a routine case. However, it may expand the capacities and safety of ESS in approaching complex deformities, tumors, and previously instrumented spines in which normal anatomy is distorted or nonstandard work angles must be employed [55]. In instrumented fusion cases, ESS may be combined with percutaneous screw placement, for which the same navigation may also be deployed to improve screw placement accuracy compared to conventional fluoroscopy. In oncological surgery, live imaging is helpful to identify the margins of tumors, allowing the surgeon to understand better when they have performed an appropriate resection. Finally, prior instrumentation may obscure bony landmarks on standard fluoroscopy, and previous spinal surgery may eliminate them; in either case, the ability to visualize instruments in 3-dimensional space may benefit the surgeon in performing precise and efficient cases (Table 1).

## 3. Case Illustrations

### 3.1. Case 1: Endoscopic L4-5 Neuroforaminal Decompression Guided by Optic Navigation

**Figure 1:**
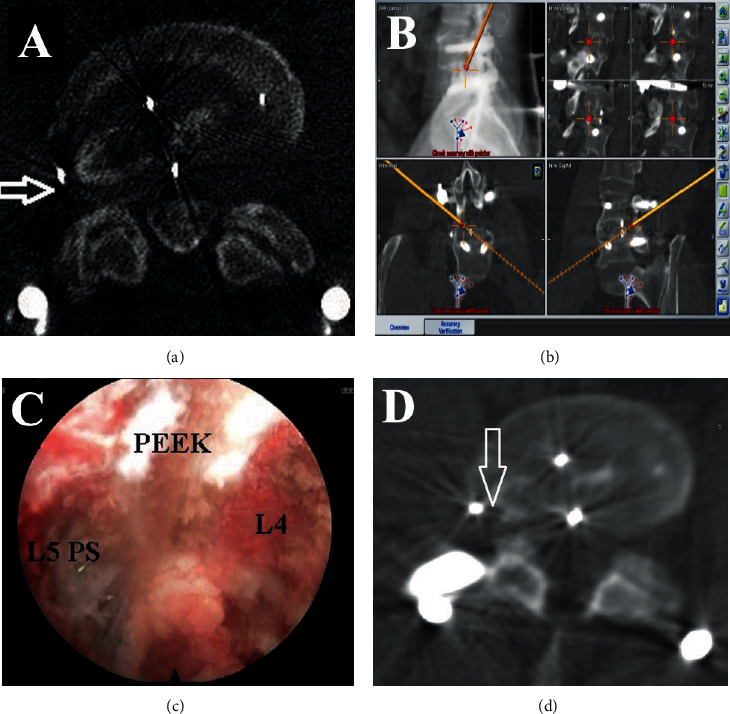
Images from optical navigation use in endoscopic spine surgery. (a) The white arrow shows the entry site to the disc space of the target level. (b) Screenshot of the navigated endoscope at Kambin's triangle. (c) Screenshot from the view of the endoscope showing the L4-5 disc space with the PEEK implant in place. (d) The white arrow points at the completed foraminotomy at the target level.

This case report was previously reported by the authors (Oyelese et al. 2018) and is represented in this narrative review article to illustrate the utility of navigation techniques in the context of ESS [49]. The patient was a healthy 49-year-old male with a prior L4-5 oblique lumbar interbody fusion (OLIF) two years before presentation for a left paracentral disc herniation. Following his OLIF, the patient experienced relief of his left lumbar radiculopathy. However, the patient then began experiencing right-sided radicular symptoms, which was thought to be due to the placed interbody cage compressing the right L4-5 neural foramen. The patient's right-sided radicular symptoms resolved with conservative management after three months. A year after the patient's index surgery, his right-sided radicular symptoms recurred. A CT myelogram demonstrated heterotopic bone in the right L4-5 foramen attributable to the patient's symptoms (Figure 1(a)).

The patient was positioned prone on the Trumpf table (Trumpf Medical, Farmington CT), prepped, and draped. The digital reference array for the Brainlab Curve® navigation system (Brainlab, Munich Germany) was placed over the skin at L5-S1 and affixed with an antimicrobial adhesive drape. Using an AIRO® intraoperative CT scanner (Brainlab, Munich, Germany), the first CT scan was obtained to allow autoregistration of the reference array to the patient anatomy for spinal navigation and CT-based 3D reconstruction the lower lumbar spine. To maintain registration accuracy, caution was taken to ensure that the reference array was not moved, which was not difficult because the incision used in the endoscopic approach was 12 cm laterally to the right, away from the midline. CT-identifiable anatomic landmarks were confirmed using a precalibrated probe registered to the optical navigation system (Figure 1(b)). A second reference array was attached to the Shrill drill (Joimax Inc., Irvine, CA), which was then registered to the navigation system. We were able to navigate the drill during the procedure with secondary confirmation from fluoroscopy. A postprocedural confirmatory CT of the L4-5 area was performed following the surgical decompression.

The procedure was done under local analgesia, and intravenous sedation with the amount of anesthetic titrated so that the patient could communicate with the surgeon throughout the procedure. The TESSYS endoscopic system (Joimax Inc., Irvine CA) was used for the procedure. A percutaneous endoscopic approach was taken for neuroforaminal decompression using an 18-gauge needle and sequential reamers to remove the ventral aspect of the superior facet and the lateral part of the heterotopic bone (Figure 1(c)). Retractors placed in Kambin's triangle then allowed for access to the neuroforamen. A drill registered with the navigation system and direct endoscopy were then utilized to maintain 360-degree visualization of the neuroforamen for successful endoscopic decompression (Figure 1(d)). The patient experienced immediate pain relief and communicated this during the surgery. As the case was performed under MAC anesthesia, an immediate postoperative exam was performed to confirm of resolution of preoperative symptoms. Patient has had continued symptom relief for six months after surgery. Greater detail regarding key endoscopic steps is described in the previously published report of this case [49].

### 3.2. Case 2: ESS Guided by Electromagnetic Navigation

**Figure 2:**
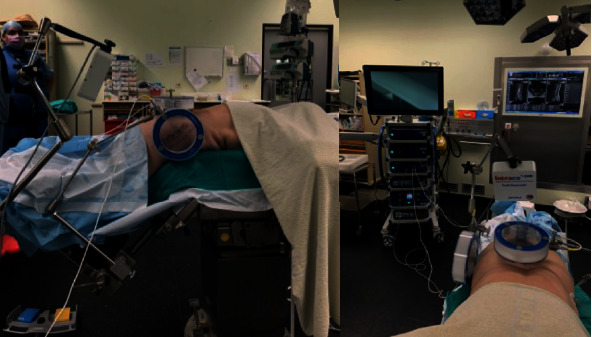
Patient installed in prone position. The two mappers and the electromagnetic field generator are fixed to the table by articulated arms. Anteroposterior and lateral mappers are placed to cover the desire working area, while the electromagnetic field generator is oriented with a 45° angle toward the working area.

**Figure 3:**
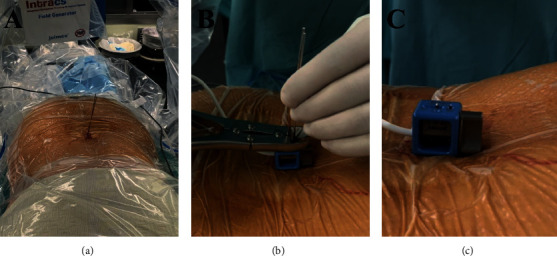
Two K-wires are fixed in a spinous process close to the working area (a). Patient tracker fixed to the K-wires and the extensions are cut to avoid electromagnetic disturbance (b and c).

**Figure 4:**
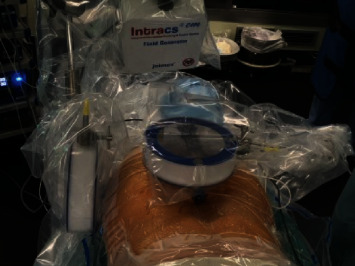
Final position of patient tracker, patient mappers, and electromagnetic field generator before fluoroscopy.

**Figure 5:**
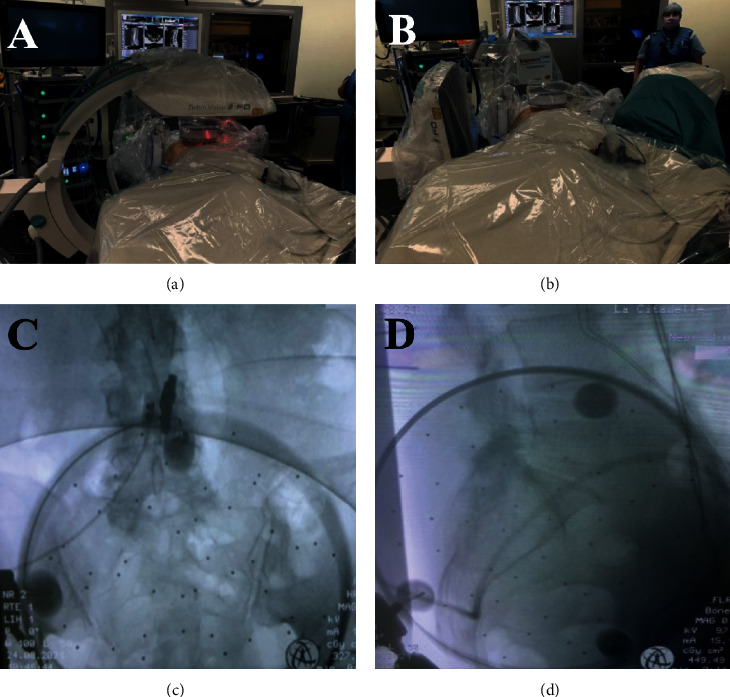
Two fluoroscopy shots are taken: one anteroposterior (a, c) and one lateral (b, d). The mappers should cover the desire navigable area.

**Figure 6:**
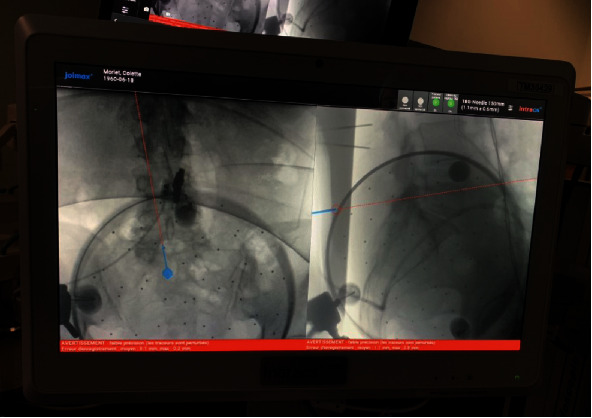
Photography of the Intracs monitor depicting the planification of the entry point after having registered the needle.

We used the Intacs®^em^ electromagnetic system (joimax®). This system works with a control unit, an electromagnetic field generator, two patient mappers with unique titanium markers footprint, one patient tracker, and sensor wires to allow instruments navigation. First, the field generator and the patient mappers are attached to the table using articulated arms (Figure 2). Two K-wires are inserted in the spinous process closest to the working area (Figure 3). The patient tracker is then fixed on those K-wires, and its cable is connected to the control unit. The extensions of the K-wires are cut close to the surface of the patient tracker. Then, the field generator is placed at a 45° angle looking to the working area. The two patient mappers are placed, one anteroposterior and one lateral, at a 90° angle from each other (Figure 4). The cables of the field generator and patient mappers are connected to the control unit. Two X-rays are taken: one anteroposterior and one lateral (Figure 5). The C-ARM and the patient's mappers are removed. The sensor wire is registered using the patient tracker. The entry point can then be navigated, and the procedure can be done with only two X-rays (Figure 6). Once the entry point is identified, the endoscopic foraminotomy and discectomy can proceed with the added benefit of a navigated scope and working channel. A small 1 cm incision is made at the planned entry point. The K-wire is introduced and targeted to Kambin's triangle. Serial dilators are introduced followed by the working channel and the endoscope. At this time, the foraminotomy can be performed with both direct visualization and 3D navigation. Once the decompression is complete, the wund is irrigated and the incision closed with stitches.

## 4. Future Directions

### 4.1. Use of Augmented Reality in Endoscopic Spine Surgery

Augmented reality (AR) is a promising new technology increasingly applied in minimally invasive spine surgery, particularly for the percutaneous placement of pedicle screws [59–62]. The technology involves a combination of virtual segmentation of anatomic features that can be registered with the patient's native anatomy and viewed as virtually superimposed upon the operative field through commercially available headsets, as well as through some advanced microscopy systems [62]. As such, augmented reality could, in theory, replace other navigation systems while circumventing the radiation-related limitations of existing navigation techniques raised above if it can be proven that AR-based systems can offer comparable precision and intraoperative reliability [63]. In an initial 2018 cadaveric study, two surgeons placed 18 thoracolumbar pedicle screws under AR assistance, with two screws breaching between 2 and 4 mm, yielding an accuracy rate of 89% [59]. In a subsequent human trial performed by the same authors with the placement of 253 pedicle screws, a 94.1% accuracy rate was achieved using AR for screw placement, comparable to accuracy rates reported when using other navigation systems in the literature [60]. One group has reported using AR-based techniques combined with ICT-Nav-guided lateral MIS approaches to the lumbar spine for cases with mixed indications, including disc herniations and spinal column tumors [64].

Clinical application of AR in MIS and ESS remains in its infancy [65]. Limited case reports and preclinical studies have examined the use of AR in percutaneous transforaminal endoscopic discectomy and endoscopic lumbar interbody fusion, sometimes in combination with other navigation techniques [66–68]. Standalone use of fully integrated AR systems for guidance in ESS is yet to be seen. Still, enormous potential exists for expanding this technique, given the success of implementing it for percutaneous pedicle screw placement [62]. Furthermore, exact radiographic registration software packages such as what would be needed for an AR-based tool are already in use across other navigation platforms [27, 62]. AR-based techniques are also helpful for clinical education and even preoperative planning, which may further improve the learning curve for surgeons seeking to implement ESS [62, 69].

### 4.2. Use of Robotic Guidance in Endoscopic Spine Surgery

Similar to other alternative navigation approaches, robotic guidance has been most robustly studied in the minimally invasive percutaneous placement of pedicle screw fixation and offers a high degree of precision for this purpose [70, 71]. Robotic guidance in percutaneous pedicle screw placement significantly decreases fluoroscopy times, suggesting it may also lower operative radiation exposure [72, 73]. Robotics also stands out as particularly easy to integrate with existing ESS approaches given that both techniques leverage tiny incisions while maintaining the ability to visualize operative anatomy directly. Robotic-assisted approaches have been studied in various ESS procedures, including laminotomy, lumbar discectomy, lumbar interbody fusion, and lumbar corpectomy [74–77]. One retrospective comparative study of robot-assisted versus only fluoroscopy-assisted percutaneous lumbar endoscopic discectomy found statistically significant reductions in fluoroscopy use, puncture-channel time, and total operative time with the robot-assisted procedure [74].

However, even when implementing robotic assistance, some alternative navigation modality is often utilized to establish a safe approach through trajectory planning in 3D [58]. Compared to electromagnetic navigation-guided procedures, which as noted above may even further minimize radiation exposure, another study found that robotic-assisted lumbar interbody fusion without electromagnetic navigation resulted in longer guide-wire insertion time and ultimately greater X-ray exposure, underscoring that robotics seems unlikely to replace other modern navigation systems in ESS entirely [78]. Furthermore, robotic assistance does not resolve the issue of confirming a safe corridor to the target foraminal pathology as other navigation modalities do. Some reports have mentioned the limited range of tools available, resorting to the traditional endoscopic technique for some critical aspects of the procedure [76]. The use of robotics in combination with advanced computer-guided navigation systems has not been studied nor have there been numerous studies comparing robotic-assisted to traditional ESS head-to-head, though there is speculation that these future directions will likely further advance spinal robotics towards broader clinical implementation [27, 79].

## 5. Conclusion

ESS expands the boundaries of MIS and is primed to disrupt current surgical practices in treating spinal pathology. Optical and electromagnetic navigation are both reasonable means of imaging guidance in endoscopic procedures with unique advantages and disadvantages. Electromagnetic navigation is the newer option and may reduce overall radiation exposure. Navigation within the discipline of ESS may assist in overcoming the limitations to the surgical technique, such as a steep learning curve and potentially disorienting surgical anatomy. Augmented reality and robotic guidance are promising technologies that continue to evolve and will likely be translated into future practice, guided by established ESS principles.

## Figures and Tables

**Table 1:** Review of the differences, advantages, and disadvantages of the different types of navigation techniques within the context of minimally invasive spine surgery.

Characteristic	Optical	Electromagnetic
Equipment	Infrared tracking camera, reference array	Control unit, an electromagnetic field generator, and specialized sensors
Improvement of the learning curve	Yes [4]	Needs more evidence
Assistance with bony landmark localization	Yes	Yes
Reference array secured to bony landmark	Yes	No
More freedom with movement of instruments		Yes, as compared to optical navigation
Radiation exposure to operating room staff as compared to conventional navigation techniques.	Potentially decreased [22, 37, 38]	Decreased [24, 25]
Improved localization of instruments, avoiding neural injury or dural tears	Yes	Yes
Advantages in nonnormal anatomy cases (tumor, deformity, etc.)	Yes [55]	Yes [55]
Interruption by ferromagnetic materials	No	Yes
Line-of-sight issues between surgical field and tracking camera	Yes	No

## Data Availability

There is no data analyzed in the present manuscript as it is a review article.
